# MiRNA-125a-5p: a regulator and predictor of gefitinib’s effect on nasopharyngeal carcinoma

**DOI:** 10.1186/1475-2867-14-24

**Published:** 2014-03-07

**Authors:** Yanyang Liu, Zhixi Li, Lu Wu, Zi Wang, Xia Wang, Yang Yu, Qian Zhao, Feng Luo

**Affiliations:** 1 Department of Medical Oncology, Cancer Center and State Key Laboratory of Biotherapy, West China Hospital of Sichuan University, 37 Guo xue Xiang Street, Chengdu 610041, Sichuan Province, China

**Keywords:** miRNA, miR-125a-5p, Nasopharyngeal carcinoma, Gefitinib

## Abstract

**Background:**

Nasopharyngeal carcinoma (NPC) is a common malignancy in China and Southeast Asia. Radiotherapy is the major treatment modality for patients with NPC, but does not always achieve fully satisfactory outcomes. Studies have shown that epidermal growth factor receptor (EGFR) is highly expressed in NPC, and EGFR-targeted treatment is expected to be a new strategy for NPC. Recently, clinical trials have shown that NPC patients have different responses to gefitinib. Thus, the identification of indicators that can regulate and predict the sensitivity of NPC to gefitinib is very valuable. MiRNAs (MicroRNAs) are closely related to cancer development. We studied miRNAs in NPC cell lines to identify those that can regulate and predict the effectiveness of gefitinib on NPC.

**Methods:**

CCK8, Annexin V-FITC assays and animal models were carried out to evaluate the inhibitory effect of gefitinib on NPC cell lines HNE-1 and HK-1. MiRNA microarrays were used to detect and compare the miRNAs expression levels in the two cells with gefitinib or not, and qRT-PCR was used to evaluate miR-125a-5p expression in NPC cells and in serum of the tumor animal models. Loss-of-function and gain-of-function experiments were taken to evaluate the effect of miR-125a-5p on gefitinib effectiveness. Western blots were used to evaluate the effect of miR-125a-5p on p53 and Her2 in HNE-1 and HK-1 cells.

**Results:**

Gefitinib inhibited two NPC cell lines proliferation in vitro and in vivo,and HNE-1 cells were less sensitive than HK-1 cells to gefitinib.MiR-125a-5p expression levels were increased by geftinib in the two cell lines and in the serum of NPC tumor bearing-mice. This phenomenon was weak in HNE-1 cells and strong in HK-1 cells. MiR-125a-5p over expression improved anti-proliferative and pro-apoptotic effects of gefitinib on the NPC cells and that miR-125a-5p down-regulation decreased those effects. MiR-125a-5p also increased p53 protein expression in HNE-1 cells, and decreased Her2 protein expression in HNE-1 and HK-1 cells.

**Conclusions:**

Our results indicate that gefitinib sensitivity and some miRNAs expressions varied in NPC cell lines. The miR-125a-5p is a possible candidate that can regulate and predict the effect of gefitinib on NPC.

## Introduction

Nasopharyngeal carcinoma (NPC) is a common malignancy in China and Southeast Asia. Radiotherapy can improve the prognosis of patients with early NPC. However, as the early symptoms are not obvious, 60-70% of patients with NPC are not diagnosed until the disease reaches an advanced stage. Concurrent radiotherapy and chemotherapy is the standard method for locally advanced NPC. Although radiotherapy technology is continuously being improved, the average 5-year survival rate of patients with NPC is only 30-40%, because the anatomical characteristics of the nasopharyngeal region limit the radiotherapydosage [1]. Thus, new clinical strategies are urgently needed to effectively treat NPC.

It is known that epidermal growth factor receptor (EGFR) play important roles in tumor proliferation, invasion, and metastasis. Studies show that EGFR is highly expressed in a variety of tumors, especially in head and neck cancer, for which the positive rate of EGFR is 80-100% [2]. Targeted therapy for NPC has made considerable progress in recent years. For instance, nimotuzumab, a monoclonal antibody that targets EGFR, has been used for the clinical treatment of NPC and achieved good efficacy [3]. However, few clinical trials have assessed the effect of NPC treated with small-molecule drugs that act by inhibiting EGFR tyrosine kinase (EGFR- Tyrosine Kinase Inhibitor). The main reason is that the mechanism of EGFR-TKI is not well understood, and the role of abnormal EGFR signaling in head and neck tumor development remains unclear. This gap in knowledge encourages more studies to ultimately develop novel therapies for NPC.

In recent years, several clinical trials have provided evidence that patients with recurrence or metastasis of advanced NPC had good endurance to gefitinib (gefitinib, Iressa), a small-molecule EGFR inhibitor, but the effect of gefitinib varies among NPC patients [4-6]. The identification of biomarkers that can predict or regulate the effect of gefitinib on NPC will help us to screen patients with NPC who are especially sensitive to gefitinib and also help to improve gefitinib effectiveness. Studies have shown that EGFR mutations are associated with gefitinib effectiveness, and EGFR gene mutation detection is a method to predict the outcome of lung cancer patients treated with gefitinib [7,8]. Although this method can identify the sites and types of EGFR mutations, many obstacles limit it from becoming a routine diagnostic sequencing method. It is difficult to obtain sufficient tumor tissues for sampling, especially as the anatomic structure of the nasopharyngeal space is limited. In addition, the majority of studies on the relationship between EGFR mutations and gefitinib have been performed in lung cancers; few studies have been published on data from NPC patients. Given this lack of data, it is difficult to predict the curative effect of gefitinib on NPC based on tumor gene mutations. Novel biomarkers would be useful for estimating gefitinib effectiveness.

MiRNAs (MicroRNAs) are small 19- to 24-nt non-coding single-stranded RNA molecules. They are ubiquitous in plant and animal cells. In animals, the vast majority of miRNAs target mRNAs by binding the 3'UTR to inhibit gene translation. They regulate growth and development and have been implicated in the development and progression of a variety of diseases [9,10]. To date, thousands of miRNAs have been found and input into a miR database [11] (miR Base). The expressional characteristics of many miRNAs are tissue specific, and this is especially true for tumors [12,13]. Many lines of evidence show that miRNAs can be direct gene therapy targets for tumors and can also be used as biomarkers to predict patient prognosis [14,15]. Recently, tumor-derived circulating miRNAs have been found in the peripheral blood of tumor patients [16]. It suggests that miRNA detection may also be a non-invasive diagnosis approach of tumor. Further research into the relationship between miRNAs and tumors will further our understanding of tumor development, improve gene therapy, and identify novel biomarkers for treatment effect and tumor prognosis.

MiRNAs are also implicated in the NPC. Zhang *et al*. found that miR-141 targeted genes such as BRD3, UBAP1, and PTEN in NPC played an important role in tumor development. Inhibition of miR-141 could affect NPC cell growth, invasion, and metastasis [17]. Sengupta *et al*. identified eight miRNAs that were differentially expressed between NPC tissues and surrounding normal tissue. One miRNA, miR-29, could affect the activity of extracellular matrix proteins to regulate tumor invasion and metastasis, and was significantly reduced in NPC [18]. Still, few studies have assessed the relationship between miRNAs and the effect of gefitinib on NPC.

In the present study, we observed that the human NPC cell line HNE-1 was relatively resistant to gefitinib, and HK-1 was relatively sensitive to gefitinib. We found that miR-125a-5p expression levels were increased by gefitinib in the two cell lines and in the serum of tumor-bearing mice, but the change degree is different. Regulation of miR125a-5p could affect the inhibition of proliferation and apoptosis of NPC cells induced by gefitinib. Collectively, our results suggest that miR-125a-5p may be a target and an indicator of gefitinib’s effects on NPC.

## Materials and methods

### Cell culture

Human nasopharyngeal carcinoma cell lines HNE-1 and HK-1 were used in this study. HNE-1 cell line was provided by Sichuan University (Chengdu, Sichuan, China) and HK-1 cell line was a gift from Professor Mu-Sheng Zeng of Sun Yat-sen University Cancer Center (Guangdong, Guangzhou, China). All the cells were cultured in RPMI1640 (Gibco, Carlsbad, CA,USA), with 10% fetal calf serum and 1% penicillin-streptomycin and at 37°C in a 5% CO_2_-humidified atmosphere.

### Cell counting kit-8 (CCK8) assay

HNE-1 and HK-1 cells were seeded in a 96-well plate at 5, 000 cells/well and allowed to adhere overnight. After treatment with gefitinib (AstraZeneca, London, UK) at various concentrations for 48 h, 10 μl CCK-8 (Beyotime, Haimen, Jiangsu, China) was added to each well. Plates were incubated at 37°C for 2 h, and then the optical density (OD) values were measured at 450 nm on a scanning multi-well spectrophotometer (Bio-Rad Model 550, CA, USA).The cell inhibitory rate was calculated by the following equation: the cell inhibitory rate = ([OD control group − OD experiment group]/OD control group) × 100*%*. All experiments were performed in triplicate and repeated three times.

### Flow cytometry assay

Cells were treated with phosphate-buffered saline (PBS) or gefitinib for 48 h and then washed with cold PBS twice, then resuspended in 1× binding buffer at a concentration of 1 × 10^5^ cells/ml. Flow cytometry was performed with an Annexin V-FITC Apoptosis Detection Kit (Beyotime) according to the manufacturer’s protocol on a Beckman Coulter Navios Flow Cytometer (Beckman Coulter, Brea, CA, USA).Results were from three separate experiments and were analyzed by au27005 Software Version: Navios (Beckman Coulter).

### Animal tumor models and treatment

We purchased 6-8-week-old athymic nude mice (SPF grade) from the Laboratory Animal Center of Sichuan University. All the studies involving mice were approved by the Institutional Animal Care and Use Committee. Athymic nude mice were inoculated with HNE-1 or HK- 1 cells (1.0 × 10^7^/0.1 ml) in the right axillary fossa. Twelve days after inoculation, when tumors were palpable, mice were randomly assigned into each group (n = 5/group). Treatments were given by gavage with normal saline (NS) for the negative control group or gefitinib (70 mg/kg/day) for the treated group every day for 14 days. Tumor volume was estimated by the formula 0.52 × length × width × width. Length and perpendicular width of tumors were measured by calipers. Tumors were weighed on an analytical balance after 14 days of treatment. Mouse blood was collected and centrifuged by 3000 rpm for 10 min to separate serum. Serum specimens were stored in −80°C for real-time reverse-transcription polymerase chain reaction (qRT-PCR) assays.

### miRNA microarray

Cell lines indicated were seeded in 6-well flat-bottomed plates and treated with PBS or gefitinib of their respective half-maximal inhibitory concentration (IC_50_) for 48 h. Then, cell miRNAs were extracted and purified by TRIzol l LS reagent (Life Technologies, Carlsbad, CA, USA), mirVana™ miRNA Isolation Kit (Applied Biosystems, Carlsbad, CA, USA) according to the manufacturers’ recommended protocols. In brief, 200 ng total RNA was dephosphorylated and labeled by using miRNA Complete Labeling and Hyb Kit (Agilent Technologies, Santa Clara, CA, USA). The purified labeled miRNA products were hybridized to Agilent Human miRNA 8 × 60 k microarrays(038166,Agilent Technologies) in a rotating hybridization oven at 10 rpm overnight at 55°C. After hybridization, the arrays were washed in Agilent Wash Buffer 1 with 0.2% SDS, 2 × SSC at 42°C for 5 min, followed by Agilent Wash Buffer 2 with 0.2 × SSC. After washing, the slides were immediately scanned with an Agilent Scan Array Express array scanner (G2565CA). The resulting images were quantified with Agilent’s Feature Extraction software (v10.7). Data were normalized and analyzed with Agilent Gene Spring software. From the microarray data, we carried out preliminary experiments to observe whether some candidates could regulate the effect of gefitinib by transfection and CCK-8 assays. We found that miR-125a-5p was a potential factor of the gefitinib’s effect on the NPC cells and it could be detected in the serum, so that we put this miRNA on the key position in this study.

### qRT-PCR

Cells and animals were treated as mentioned above. Then, miRNAs of cells and serum were obtained and purified with TRIzol l LS reagent (Life Technologies), mirVana™ miRNA Isolation Kit (Applied Biosystems), or HiPure Serum/Plasma miRNA kits (Mage, Guangdong,Guangzhou, China) in accordance with the manufacturers’ instructions. The expression levels of miRNAs were confirmed with a SYBR-based qRT-PCR using individual miRNA-specific primers. For reverse transcription (RT) reactions, 100 ng total RNA from each cell or total RNA from each 250 μl serum specimen mixed with 5 μl 1nM cel-miR-39 was used in each reaction and mixed with the RT primer. The RT reaction was carried out at 37°C for 60 min, 70°C for 10 min. After the RT reaction, 1.0 μl cell cDNA products or 3.0 μl serum cDNA products were used in the each PCR reaction (10 μl) along with miR-125a-5p primers. The PCR reaction was conducted at 95°C for 3 min followed by 40 cycles of 95°C for 10 s, 60°C for 20 s and 70°C for 1 s in a Bio-Rad CFX96 Sequence detection system (Bio-Rad, Hercules, CA, USA). Specific RT primers (Ribobio, Guangdong, Guangzhou, China) and miR-125a-5p primers (MIMAT0000443, Ribobio) were used to quantify the expression of miR-125a-5p.Samples of cells were normalized to U6 snRNA (MQP-0202, Ribobio), and samples of serum were normalized to cel-miR-39 (Ribobio) as indicated. The qRT-PCR experiments were performed in three independent experiments. Data are presented as fold differences relative to U6 snRNA or cel-miR-39 based on the equation RQ = 2^−ΔΔCT^.

### Transfection

All transfections of HNE-1 and HK-1 cells were carried out with Lipofectamine2000 (Life Technologies) according to the manufacturer’s instructions. Oligo miRNA–negative control: (FAM-oligonucleotides) (sense-5p:5′-CAGUACUUUUGUGUAGUACAA-3′), oligo miRNA-125a-5p inhibitor(2′–O-methyl oligonucleotides) (sense-5p:5′-UCACAGGUUAAAGGGUCUCAGGGA-3′) and Oligo miRNA-125a-5p mimic (2′–O-methyl oligonucleotides) (sense-5p: 5′-UCCCUGAGACCCUUUAACCUGUGA-3′) were synthesized by Integrated DNA Technologies (GenePharma, Shanghai, China). Twenty-four hours before transfection, HNE-1 or HK-1 cells were plated in 6-well dishes for RNA extraction, protein extraction. Oligo miRNA-negative control (100 nM), or oligo miRNA-125a-5p inhibitor (100 nM) or oligo miRNA-125a-5p mimic (100 nM) was transfected into HNE-1 or HK-1cells. RNA was collected 24 h after transfection and protein was collected 48 h after transfection, respectively.

### Western blot

Each cell line was seeded at about 2 × 10^5^cells per well in 6-well plates and then transfected with miRNA oligos as mentioned above when cells were be 50-70% confluent. After 48 h, cells were lysed with radio immunoprecipitation assay (RIPA) lysis buffer (Beyotime). Then sample buffer containing suspended proteins was centrifuged at 12,000 rpm for 15 min, and the supernatant was collected. A total of 30 μg of each protein sample was loaded in each well and separated with 10% SDS-PAGE. The proteins in the gel were electroblotted onto PVDF membranes (Millipore, Billerica, MA, USA) with wet blotting. Membranes were incubated in blocking buffer (1× Tris-buffered saline [TBS], 0.1% Tween-20, and 5% w/v dry nonfat milk) for 1 h at room temperature, and then membranes were incubated with anti-p53 DO-1 (1:300, Santa Cruz, Dallas, Tex, USA), or anti-Her2 29D8 (1:300, Cell Signaling Technology, Beverly, MA, USA), or anti-β-actin (sc-47778, 1:1000, Santa Cruz) for overnight at 4°C, followed by incubation with an appropriate peroxidase-conjugated secondary antibody at room temperature for 1 h. Reactive bands were detected by enhanced chemiluminescence (Millipore).

### Statistical analysis

Data are expressed as mean ± SD. Statistical Package for the Social Science (SPSS) version 16.0 (Chicago, IL, USA) was used for statistical analysis. Statistically significant differences between two groups were tested by performing unpaired Student’s t tests. Results were considered statistically significant at *P* < 0.05.

## Results

### NPC cell lines exhibited differential sensitivity to gefitinib

**Figure 1:**
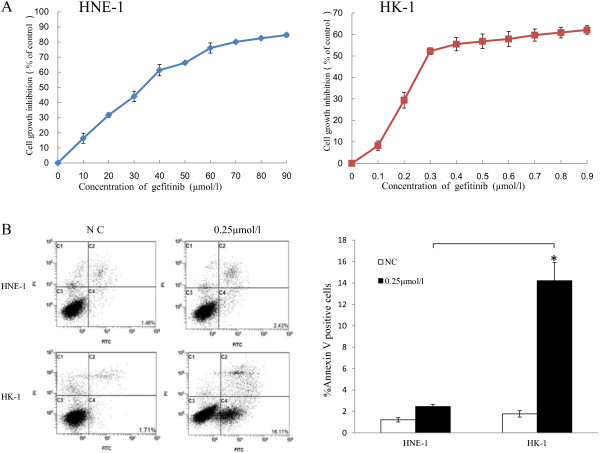
**NPC cell lines had different sensitivities to gefitinib. (A)**. Gefitinib inhibited the proliferation of NPC cell lines HNE-1 and HK-1 in vitro, as assayed by CCK-8. The IC_50_ values of HNE-1 and HK-1 for gefitinib were 35 μmol/l and 0.25 μmol/l, respectively. **(B)**. Flow cytometry results showing that 0.25 μmol/l gefitinib significantly increased more apoptosis in HK-1 cells compared with HNE-1 cells (*P* < 0.05). The results are from three independent experiments and are expressed as mean ± SD.* *P* < 0.05.

**Figure 2:**
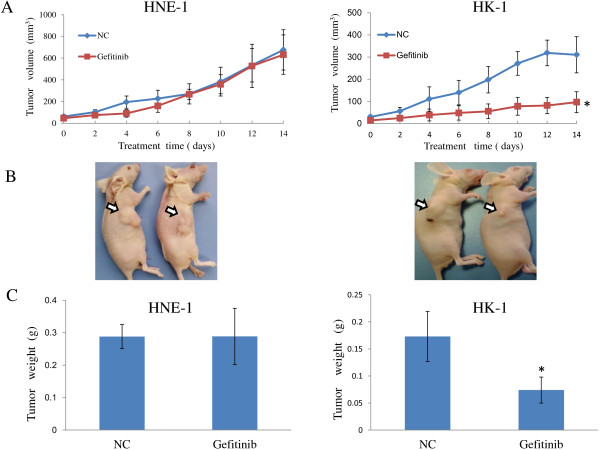
**Gefitinib-mediated inhibition of tumor growth.** Mice were treated by gavage with NS or gefitinib (70 mg/kg/day) every day for 14 days. **(A)**. Antitumor efficacy of gefitinib in HNE-1 (n = 5/group) and HK-1 (n = 5/group) models. The results are expressed as the mean volume ± SD. **(B)**. Two representative athymic nude mice from the NS-treated and gefitinib-treated groups are shown. **(C)**. Antitumor efficacy of gefitinib presented by tumor weight in the two models. The results are expressed as the mean weight ± SD.* *P* < 0.05.

In this experiment, gefitinib showed a dose-dependent inhibitory effect on the NPC cell growth. However, different NPC cell lines showed variable sensitivity. As shown in Figure 1, although gefitinib could inhibit the proliferation both of HNE-1 and HK-1 cells in vitro, the IC_50_ values of HNE-1 and HK-1 for gefitinib were approximately 35 μmol/l and 0.25 μmol/l, respectively. The Annexin V-FITC assay (Figure 1B) also showed that the number of apoptotic cells was more obviously increased after treatment with 0.25 μmol/l gefitinib in HK-1 group compared with HNE-1 group (*P* < 0.05). The results suggested that HK-1 cells are more sensitive to gefitinib than HNE-1 cells. Animal treatment experiments produced similar results. The established HNE-1 and HK-1 tumor models were used to estimate the effect of gefitinib on mouse tumor burden. The treatment regimens were performed as described above in the Materials and Methods. As shown in Figure 2, the tumor volume of the gefitinib-treated HK-1 model was limited during 14 days of treatment with 70 mg/kg/day gefitinib (*P* < 0.05). After treatment, the tumor weights in the HK-1 model of the control and treated groups were (0.173 ± 0.05 g) vs. (0.074 ± 0.024 g) (*P* < 0.05). Notably, the same dosage of gefitinib did not significantly inhibit tumor development in HNE-1 tumor-bearing mice. Taken together, these in vitro and in vivo results indicate that HNE-1 cells exhibit relative resistance to gefitinib compared to HK-1 cells.

### miR-125a-5p expression levels were increased by gefitinib in NPC cells and in the serum of tumor-bearing mice

**Figure 3:**
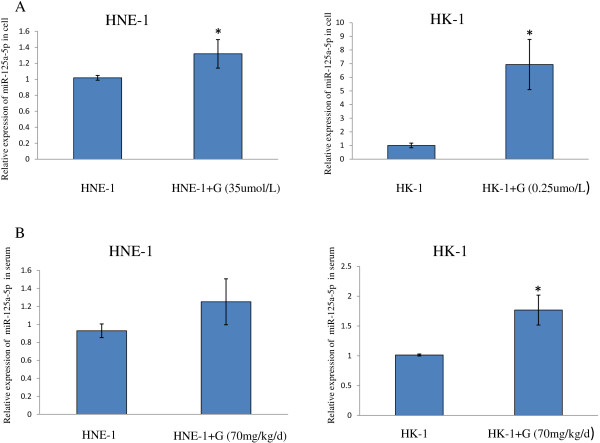
**miR-125a-5p expression levels were increased by gefitinib in NPC cells and in the serum of tumor-bearing mice. (A)**. The relative expression levels of miR-125a-5p were increased by geftinib in HNE-1 cells and in HK-1 cells (*P* < 0.05). The results were representative of three individual experiments. **(B)**. The relative expression levels of miR-125a-5p in serum were higher in gefitinib-treated groups than those in NC groups (*P* < 0.05). The results are expressed as mean ± SD. Serum specimens were from tumor-bearing mice indicated in the animal treatment experiment.* *P* < 0.05.

MiRNAs play important roles in tumor development and have good application prospects for cancer diagnosis, prognosis prediction, and genetherapy [14,15]. Some studies have reported that miRNAs can regulate the efficacy of chemotherapy drugs and molecule-targeted drugs [19]. They may become a novel type of biomarker to predict and regulate chemotherapy. Thus, we took miRNA microarrays to detect the composition of miRNAs in NPC cells and evaluate the changes of miRNAs expressions induced by gefitinib treatment. From the microarray results we found that miR-125a-5p is an interesting one. Firstly, as shown in Figure 3, qRT-PCR assays demonstrated that miR-125a-5p expression levels were increased after treatment with gefitinib both in vitro and in the serum. More notable this phenomenon was weak in HNE-1 cells and strong in HK-1 cells.

### miR-125a-5p affected gefitinib effectiveness in HNE-1 and HK-1 cells

**Figure 4:**
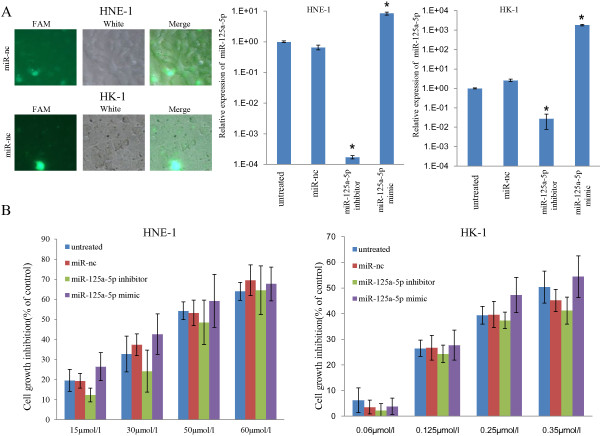
**miR-125a-5p could mediate the anti-proliferation effect of gefitinib on NPC cells. (A)**. Using oligo-miR-nc (FAM) as an example, a FAM reporter assay confirmed that the miRNA oligos used in this study were successfully transfected into HNE-1 and HK-1 cells. qRT-PCR revealed that the relative quantities of miR-125a-5p were decreased in cells transfected with oligo-miR-125a-5p inhibitor (*P* < 0.05), and miR-125a-5p was increased in cells transfected with oligo-miR-125a-5p mimic (*P* < 0.05). **(B)**. CCK-8 assay showed that the anti-proliferative effect of gefitinib on HNE-1 and HK-1 cells was weakened by transfection with oligo-miR-125a-5p inhibitor and promoted by transfection with oligo-miR-125a-5p mimic. The results are representative of three individual experiments.* *P* < 0.05.

**Figure 5:**
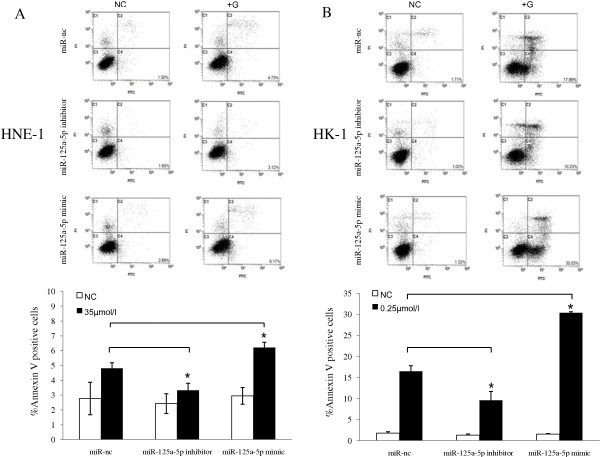
**miR-125a-5p affected gefitinib-induced NPC cell apoptosis.** Flow cytometry revealed that compared with control groups, the apoptosis rate of gefitinib-treated HNE-1 cells **(A)** and HK-1 cells **(B)** was decreased in cells transfected with olig-miR-125a-5p inhibitor (*P* < 0.05)and increased in cells transfected with oligo-miR-125a-5p mimic (*P* < 0.05). The results are representative of three individual experiments.* *P* < 0.05.

Secondly, to directly observe the function of miR-125a-5p in the effectiveness of gefitinib on NPC, we performed loss-of-function and gain-of-function experiments in which we decreased and increased the quantities of miR-125a-5p with oligo-miR-125a-5p inhibitor (*P* < 0.05) and oligo-miR-125a-5p mimic (*P* < 0.05), respectively (Figure 4A). Then we assessed cell proliferation with CCK-8 assays. HNE-1 and HK-1 cells (Figure 4B) showed similar tendencies; compared with the control groups, overexpression of miR-125a-5p by oligo-miR-125a-5p mimic improved the anti-proliferative effect of gefitinib and limitation of miR-125a-5p expression by oligo-miR-125a-5p inhibitor weakened the anti-proliferative effect of gefitinib. Annexin V-FITC assay by flow cytometry (Figure 5) further demonstrated that oligo-miR-125a-5p mimic clearly enhanced HK-1 cell apoptosis (*P* < 0.05) induced by gefitinib, and oligo-miR-125a-5p inhibitor significantly decreased gefitinib-induced HK-1 cell apoptosis (*P* < 0.05). Similar results were observed in HNE-1 cells, though the change was less obvious when compared with HK-1 cells. Taken together, these results indicate that miR-125a-5p could regulate gefitinib’s anti-proliferative and pro-apoptotic effects on NPC cells.

### miR-125a-5p affected p53 and Her2 expression

**Figure 6:**
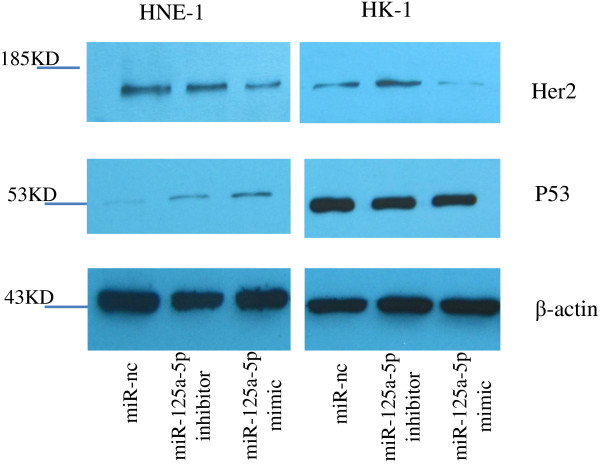
**miR-125a-5p mediated the expression of p53 and Her2 proteins in NPC cells.** Western blot analysis confirmed that p53 protein expression was lower in HNE-1 cells than in HK-1 cells. After transfection with oligo-miR-125a-5p mimic, p53 protein expression was increased in HNE-1 cells compared with the control group. Her2 protein expressions were decreased in both cell lines following transfection with oligo-miR-125a-5p mimic in HNE-1 and HK-1 cells. Her2 protein expressions in HK-1 cells were increased after transfection with oligo-miR-125a-5p inhibitor.

Studies have shown that p53 and Her2 are targets of miR-125a-5p, and existing evidence suggests that the protein expression levels of p53 and Her2 are related to the effectiveness of EGFR inhibitors on tumors [20-25]. In the present study, we detected p53 and Her2 protein levels by western blot. Results shown in Figure 6 suggest that p53 protein expression was lower in HNE-1 cells than in HK-1 cells. MiR-125a-5p mimic increased p53 protein expression in HNE-1 cells but not in HK-1 cells. We found that miR-125a-5p mimic decreased Her2 protein expression in both HNE-1 and HK-1 cells, and miR-125a-5p inhibitor enhanced Her2 protein expression in HK-1 cells. Data suggest that miR-125a-5p may target Her2 to exert the effect of gefitinib against NPC cells. As mentioned above, regulation of p53 and Her2 by miR-125a-5p may be the potential connection between this miRNA and gefitinib effectiveness.

## Discussion

In the present study, we found that although gefitinib inhibited NPC cell proliferation in vitro and in vivo, sensitivity varied between the two NPC cell lines. Specifically, HNE-1 and HK-1 cells were relatively resistant and sensitive to gefitinib, respectively. We also found that miRNAs expression levels were different between the two cell lines. Further, the selected miR-125a-5p regulated the effect of gefitinib on HNE-1 and HK-1 cells, and increased p53 protein expression in HNE-1 cells and suppressed Her2 protein expression in HNE-1 and HK-1 cells.

The CCK-8 assay results showed that the IC_50_ value of HK-1 for gefitinib (about 0.25 μmol/l) was the lowest compared with other NPC cell lines that had IC_50_ values ranging from 20–40 μmol/l. Similar results were reported by Ma *et al*. [26]. We employed HNE-1 and HK-1 cells in the present study based on their IC_50_ values (Figure 1). Animal treatment experiments further demonstrated that HNE-1 was relative gefitinib resistant and HK-1 was relative gefitinib sensitive (Figure 2). We then performed miRNA microarrays to detect and compare miRNA composition in HNE-1 and HK-1 cells. Some miRNAs were differentially expressed between the two cell lines or changed after gefitinib treatment. We carried out preliminary experiments to investigate whether some candidate miRNAs associated with tumor development, including miR-483-5p, miR-196a, miR-125a-5p, etc., could mediate the effect of gefitinib on NPC cells. We found that one miRNA, miR-125a-5p, seemed to be the most likely candidate. We performed loss-of-function and gain-of-function experiments to decrease miR-125a-5p quantities by transfecting cells with oligo-miR-125a-5p inhibitor and increase miR-125a-5p quantities by transfecting with oligo-miR-125a-5p mimic (Figure 4A). We found that miR-125a-5p overexpression improved anti-proliferative and pro-apoptotic effects in gefitinib-treated NPC cells and that miR-125a-5p down-regulation decreased those effects (Figures 4B, and 5). Western blotting demonstrated that p53 protein expression was increased in HNE-1 cells transfected with oligo-miR-125a-5p mimic. Her2 protein expression levels were reduced both in HNE-1 and HK-1 cells transfected with oligo-miR-125a-5p mimic.

In addition, in vitro and in vivo qRT-PCR results (Figure 3) both showed that miR-125a-5p expression levels increased significantly in HK-1 and slightly in HNE-1 after gefitinib treatment. Notably, compared with HNE-1, HK-1 cell lines and HK-1 tumor-bearing mice were more sensitive to gefitinib treatment. Thus, the data suggested it may be a way to predict gefitinib’s effects on NPC by estimating the change degree of miR-125a-5p after gefitinib treatment. The higher increased miR-125a-5p means the better drug effect. Especially, the change can be reflected by serum miRNA, making it seems to be more valuable [27,28].

Some potential mechanisms may explain gefitinib’s effect on NPC cells promoted by miR-125a-5p. Based on the target proteins of miR-125a-5p that have been reported, we carried out western blotting to detect p53 and Her2 proteins. Both are related to EGFR inhibitor and are important factors associated with apoptosis. Jiang *et al*. [20] reported that miR-125a-5p could induce apoptosis in A549 cells in a p53-dependent manner by increasing p53. Our results (Figure 6) indicated that p53 levels were lower in HNE-1 cells compared to HK-1 cells. So, it is possible that low p53 expression limited miR-125a-5p’s function and rendered HNE-1 cells gefitinib resistant. However, miR-125a-5p mimic only increased p53 protein levels in HNE-1 cells; no obvious change was observed in HK-1 cells following miRNA oligos transfection in the present study. The impact of miR-125a-5p on p53 protein also remains controversial [22]. Thus, there were still some conflicting observations about miR-125a-5p, p53 and gefitinib required clarification. In addition, other lines of evidence have shown that miR-125a-5p could inhibit Her2 protein levels in the breast cancer cell line SKBR3 [23] and can induce apoptosis in the gastric cancer cell line NUGC4 by suppressing Her2 protein levels [24]. Studies suggest that there are connections among Her2 and EGFR [29]. Her2 is suggested as a potential target for EGFR inhibitors [25]. Our results showed that miR-125a-5p mimic partly suppressed Her2 protein expression in HNE-1 and HK-1 cells, and miR-125a-5p inhibitor increased Her2 protein expression in HK-1 cells. Therefore, it is possible that miR-125a-5p assisted gefitinib by suppressing Her2 protein, although more studies are needed to clarify the precise mechanisms.

In conclusion, this study demonstrates that sensitivities to gefitinib were different between two NPC cell lines, and miR-125a-5p as a potential candidate that could regulate and predict gefitinib’s effect on NPC. Our work suggests that further studies on miRNAs could help to find novel therapeutic targets and curative effect predictors for NPC.

## Abbreviations

NPC: Nasopharyngeal carcinoma; CCK-8: Cell counting kit-8; FITC: Fluorescein isothiocyanate; qRT-PCR: Real-time reverse-transcription polymerase chain reaction; miRNA: microRNA; IC50: Half-maximal inhibitory concentration; FAM: Carboxyfluorescein.

## Competing interests

The authors declare that they have no competing interests.

## Authors’ contributions

FL and YYL conceived and designed the experiments. YYL, ZL and WL performed the experiments. YYL, ZXL and LW analyzed the data. YYL, LW, ZXL, ZW, XW, and FL contributed reagents/materials/analysis tools. YYL wrote the paper. All authors read and approved the final manuscript.
